# Compressive Strength Enhancement of Vertically Aligned Carbon Nanotube Forests by Constraint of Graphene Sheets

**DOI:** 10.3390/ma10020206

**Published:** 2017-02-21

**Authors:** Chih-Chung Su, Ting-Xu Chen, Shuo-Hung Chang

**Affiliations:** Department of Mechanical Engineering, National Taiwan University, Taipei 10617, Taiwan; r92522629@ntu.edu.tw (C.-C.S.); tim79310@gmail.com (T.-X.C.)

**Keywords:** graphene, vertically aligned carbon nanotube forests, 3D structure, mechanical properties, nanoindentation

## Abstract

We fabricated a 3D sandwich hybrid material composed of graphene and vertically aligned carbon nanotube forests (VACNTs) using chemical vapor deposition. The graphene was first synthesized on Cu foil. Then it was transferred to a substrate which had a pre-deposited catalyst Fe film and a buffer film of Al_2_O_3_ for the growth of VACNTs. The VACNTs were grown underneath the graphene and lifted up the graphene. The graphene, with its edges anchored on the Al_2_O_3_, provided a constrained boundary condition for the VACNTs and hence affected the growth height and mechanical strength of the VACNTs. We prepared three groups of samples: VACNTs without graphene, VACNTs with graphene transferred once (1-Gr/VACNTs), and VACNTs with graphene transferred twice (2-Gr/VACNTs). A nano-indentation system was used to measure the reduced compressive modulus (Er) and hardness (H). The Er and H of Gr/VACNTs increased with the number of transfers of the anchored graphene. The 2-Gr/VACNTs had the largest Er and H, 23.8 MPa and 912 KPa, which are 6.6 times and 5.2 times those of VACNTs without the anchored graphene, respectively. In this work, we have demonstrated a simple method to increase the mechanical properties and suppress the height of VACNTs with the anchored graphene and number of transfers.

## 1. Introduction

Carbon nanotubes (CNTs) and graphene, both of which are carbon-based nanomaterials, have excellent mechanical, thermal, and electrical properties, so they are currently popular topics in scientific research and nanotechnology [1,2,3,4,5]. The most popular growth method of graphene and CNTs is chemical vapor deposition (CVD) [6,7,8,9]. Additionally, Hart et al. reported a method to control the sharp and the structure of carbon nanotube forests by a pre-fabricated mold [10]. However, the method is complex and expensive. In recent years, several theoretical and experimental reports have increased interest in 3D hybrid nanostructures combining graphene and CNT forests, including the fabrication methods of the structures [11,12,13,14,15]. These reports have shown that the 3D hybrid material has a wide range of potential applications, such as microsupercapacitor [12] and hydrogen storage [16]. The 3D hybrid material was fabricated by first growing the graphene on the substrate, after which the catalyst Fe film and buffer layer Al_2_O_3_ were deposited on top of the as-grown graphene to grow vertically aligned carbon nanotube carpets (VACNTs) using the CVD method [11,12]. Paul et al. reported a transfer method for fabricating the 3D hybrid structure in which the graphene was on the top of the hybrid structure [15]. However, most previous reports have noted that the electrical properties and hybrid structure of substrate/graphene/VACNTs limit their application. To date, no discussions of the mechanical properties of the 3D hybrid material have been published. This work focuses on the direct growth of the 3D structure of substrate/VACNTs/graphene. The mechanical properties of the 3D material with the graphene transferred once and twice are also discussed.

## 2. Materials and Methods

**Figure 1:**
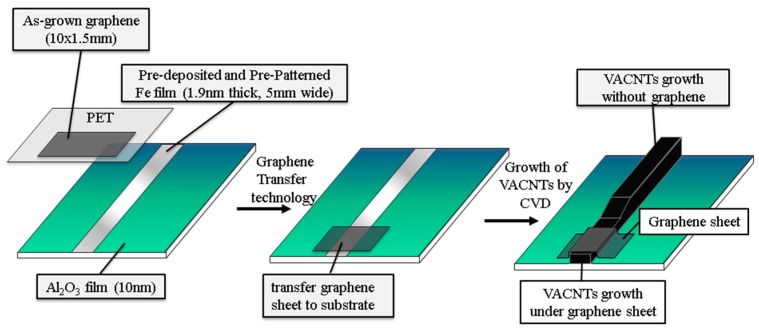
Fabrication procedure of graphene/vertically aligned carbon nanotubes (VACNTs) hybrid structure. The catalyst Fe film is for growth of the VACNTs.

In this work, a 3D hybrid structure with the VACNTs sandwiched between the substrate and graphene was constructed. The graphene was synthesized with a three-temperature-zone CVD (TTZ-CVD) process. The as-grown grapheme was first transferred to the substrate, after which the VACNTs were synthesized by CVD method to form the hybrid structure of substrate/VACNTs/graphene. As-cleaned Cu foil (99.8% purity) was first placed on a quartz boat and inserted into a TTZ-CVD furnace, where it was heated to 1000 °C at 30 min under H_2_ of 30 sccm and argon (Ar) of 600 sccm. The Cu foil was annealed for 30 min at that temperature to remove oxides of Cu in H_2_ and Ar atmosphere. The mixed reaction gas of CH_4_, H_2_, and Ar was admitted into the CVD at flow rates of 10, 30, and 600 sccm for 5 min to grow the graphene sheet. Afterward, the CH_4_ flow was stopped and the sample was rapidly cooled to room temperature. Next, the as-grown graphene sheet was transferred onto the target substrate with a clean-lifting transfer technique utilizing electrostatic force [17]. The transfer procedure began with the as-grown graphene/Cu foil attached on the PET (poly ethylene terephthalate) by electrostatic force. After a pressing process to increase the attachment between the Cu foil and PET, the sample was immersed into Cu etchant (98% sodium persulfate) and heated to 60 °C to etch the Cu foil. The sample was then rinsed with IPA and DI water to remove residual etchant. The PET with the attached graphene was pressed, and the PET was then removed to transfer the graphene onto the target substrate, which had a pre-deposited 10 nm buffer layer of Al_2_O_3_ and a 1.9 nm catalyst Fe film. It was defined the deposited zone by the tape as shadow mask pattern and released it after the deposition of buffer layer and catalyst film. In addition, the attached graphene on PET was transferred onto one side of the pre-patterned Fe film for comparison of the mechanical properties with or without graphene, as shown in Figure 1. Subsequently, the target substrate was placed into the quartz tube and heated to 750 °C under a 600 sccm flow of Ar gas. To grow the VACNTs, a H_2_ flow of 400 sccm and C_2_H_4_ gas was supplied as the carbon source at a flow rate of 40 sccm for 60 min at 750 °C. The growth processes were conducted under 1 atm. The mechanical properties of the 3D hybrid structure of substrate/VACNTs/graphene were experimentally measured with a nanoindentation instrument.

## 3. Results and Discussion

**Figure 2:**
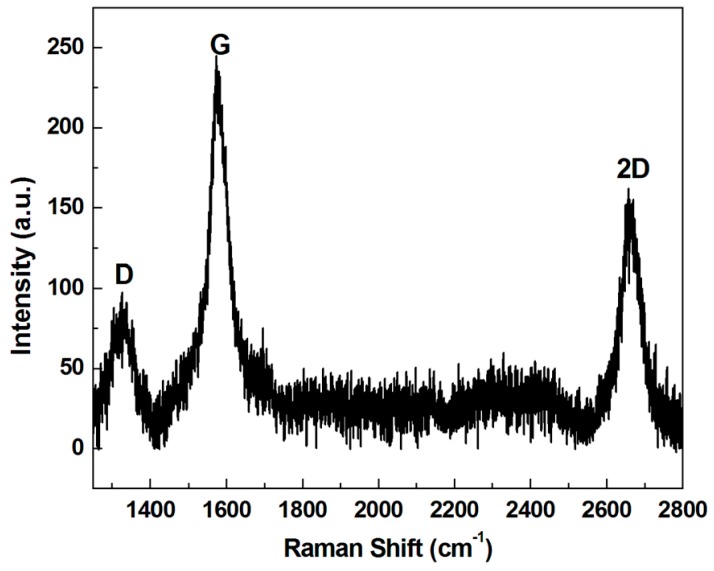
Raman spectrum of as-grown graphene on Cu foil.

**Figure 3:**
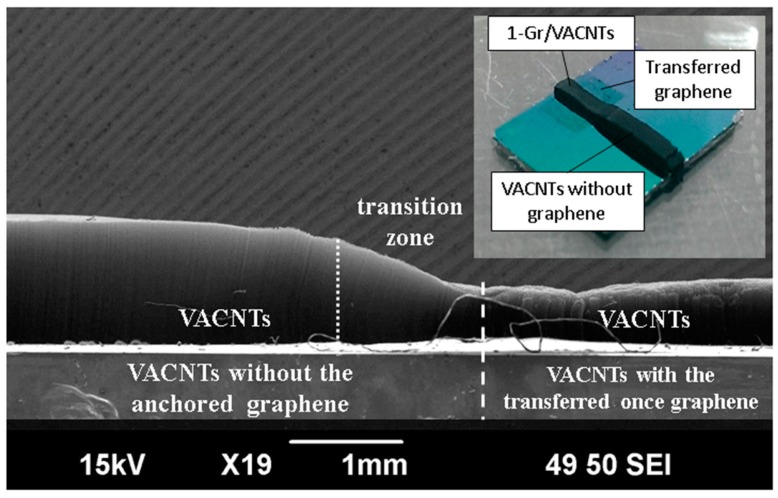
SEM (scanning electron microscope) side-view image of the 1-Gr/VACNTs with an inset showing the optical image.

Figure 2 shows the Raman spectrum of the as-grown graphene. The two main peaks corresponded to the G band (~1580 cm^−1^) and 2D band (~2700 cm^−1^) of the graphene [18]. The peak intensity of the 2D band peak was lower than that of the G band, with a peak intensity ratio of I_2D_/I_G_ ~0.67, and the full width at half-maximum of the 2D band peak was 63.1 cm^−1^. The results indicated few or more layers of graphene [19,20]. A scanning electron microscope (SEM) image is presented in Figure 3. The sample was 1 graphene sheet transferred onto the target substrate. The height of the 1-Gr/VACNTs was 509 um, lower than that of VACNTs of 1070 um without the anchored graphene. Interestingly, the morphology of VACNT was affected at the boundary zone (i.e., transition zone) due to the Van der Waals force of CNTs [21], which resulted in smooth increasing the height of VACNTs at the interface.

**Figure 4:**
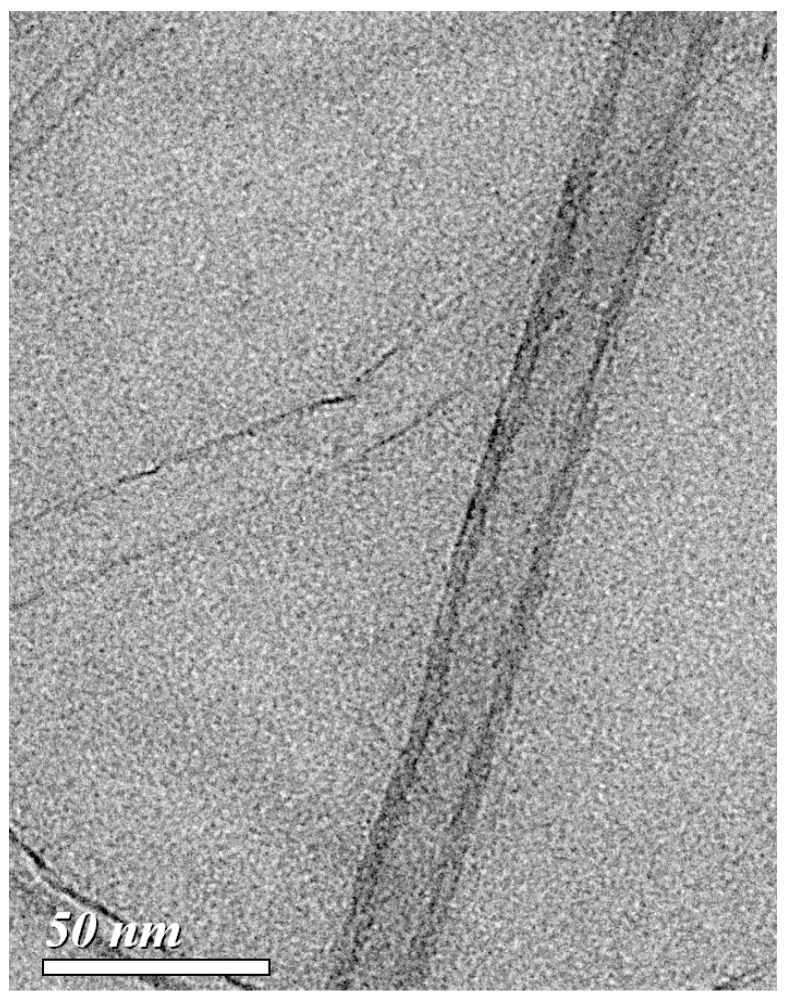
TEM (transmission electron microscope) image of multi-wall CNT.

**Figure 5:**
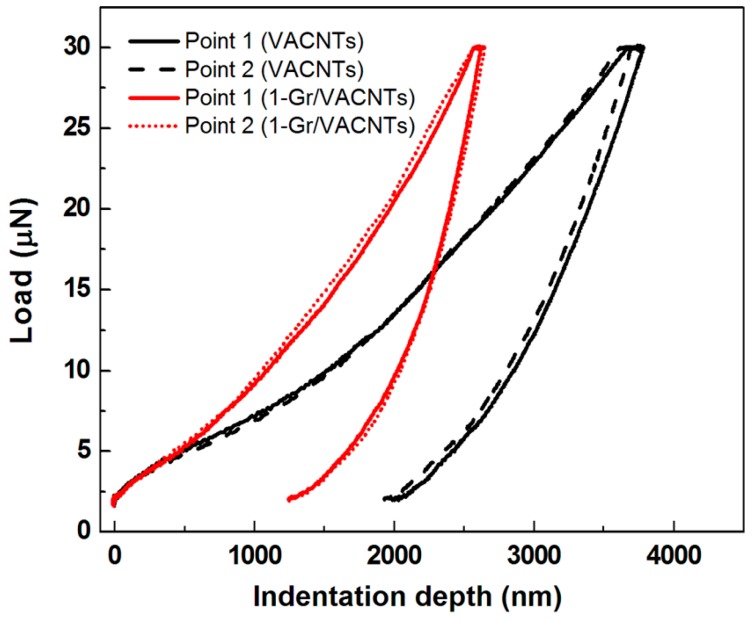
The load-depth curves of VACNTs with and without the anchored grapheme.

From the VACNTs growth process, Figure 4 shows the type of VACNTs is the multi-wall CNTs [22]. The mechanical properties of the VACNTs and 1-Gr/VACNTs (i.e., VACNTs with the graphene transferred once) were measured with a nanoindentation system (TI 950 TriboIndenter, Hysitron, Minneapolis, MN, USA). A standard Berkovich indenter was used to indent the top surfaces of the VACNTs and 1-Gr/VACNTs with a 30 µN load on the sample surface for 5 s. The slope of the unloading curve was used to determine the reduced modulus (Er) and hardness (H) of the samples according to the theory proposed by Oliver and Pharr [23]. The indentation depth of 1-Gr/VACNTs was less than that of the VACNTs sample, as shown in Figure 5, indicating that the hardness of the Gr-VACNTs was greater than that of the VACNTs. To verify the repeatable results of the nanoindentation measurement, two points were measured. The measurement results of Er and H are listed in Table 1, where it can be seen that the Er and H of 1-Gr/VACNTs were higher than those of the VACNTs. The Er and H of the 1-Gr/VACNTs were 3.633 MPa and 277 KPa, 2 times and 1.9 times those of the VACNTs, respectively. These differences indicated that the anchored graphene strengthened the mechanical properties of the VACNTs. The anchored graphene was similar to an elastic sheet constraining the growth of VACNTs, resulting in compression and a greater density of VACNTs, which enhanced the mechanical properties of the VACNTs. In addition, Wardle et al reported the densification of CNTs enhanced the mechanical properties with the increasing volume fraction of the different diameter CNTs [24].A similar effect may have occurred in this work, resulting in the greater Er and H of 1-Gr/VACNTs. The effect of pre-compression of the VACNTs suggests the graphene constraint enhanced to increase the Van der Waals force of CNTs.

**Figure 6:**
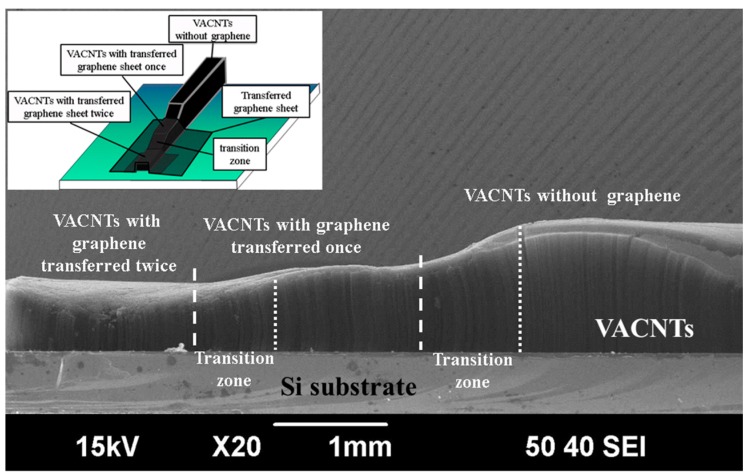
SEM images of the side view of the Gr/VACNTs, with an inset showing a sample with different layers of anchored grapheme.

**Figure 7:**
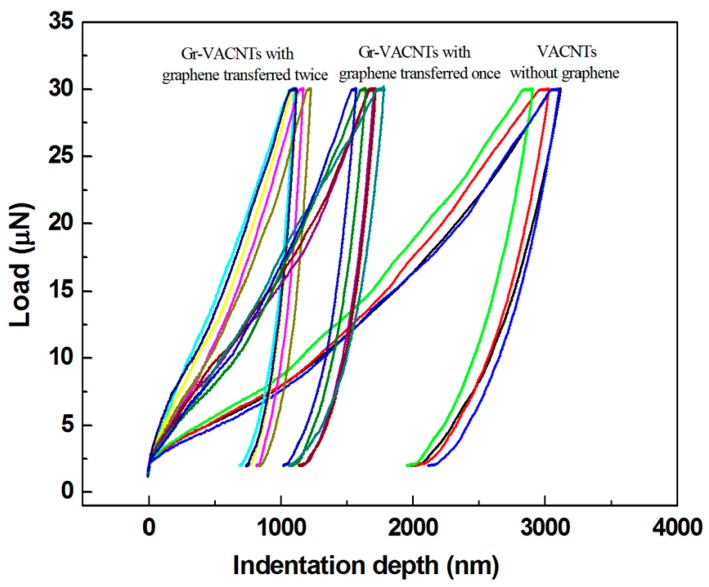
The load-depth curves of 1-Gr/VACNTs, 2-Gr/VACNTs and VACNTs.

**Figure 8:**
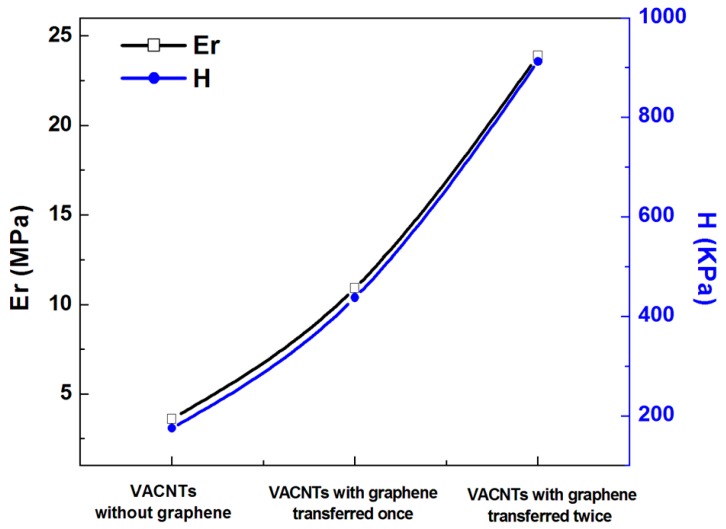
The Er and H with the graphene transferred different numbers of times.

To further examine the constraining effect of the multi-layer graphene on the mechanical properties, the graphene sheets were transferred once and twice onto different areas of the substrate and VACNTs were grown, as shown in the inset of Figure 6. Figure 6 is an SEM image of 2-Gr/VACNTs, 1-Gr/VACNTs, and VACNTs, which respectively had heights of 600 um, 740 um, and 1140 um. The height of the VACNTs with graphene transferred twice was lower than those with graphene transferred once and graphene not transferred. As can be seen from Figure 7, the Gr/VACNTs with graphene transferred twice had greater strength. The highest Er and H were respectively 6.6 times and 5.2 times those of VACNTs without the anchored graphene due to the compression and greater density of VACNTs, as shown in Figure 8. However, when the number of transfers of the anchored graphene was increased further, no VACNTs grew on the substrate. The anchored graphene layers were similar to a graphite film blocking the Fe catalysts and restrained the growth of the VACNTs. In addition, the height of the VACNTs was affected by the number of transfers of the anchored graphene.

## 4. Conclusions

In this work, a 3D hybrid nanostructure was fabricated with graphene and VACNTs. The mechanical properties of the Gr/VACNTs increased with the number of transfers of the anchored graphene. The height of the VACNTs was affected by the number of times the graphene was transferred. This simple method could be used to fabricate a high strength nano-material. In addition, VACNTs and graphene are the excellent performance of thermal interface materials. This may warrant new applications for the polishing [25] and micro-mold [26] which take advantage of high mechanical strength and greater heat dissipation.

## Figures and Tables

**Table 1:** Reduced modulus (Er) and hardness (H) of VACNTs (vertically aligned carbon nanotube) and 1-Gr/VACNTs.

Material	Reduced Modulus Er (Mpa)	Surface Hardness H (Kpa)
VACNTs	1.766	149
1-Gr/VACNTs	3.633	277
